# In the national epidemiological bulletins – a selection from recent issues

**DOI:** 10.2807/1560-7917.ES.2017.22.26.30559

**Published:** 2017-06-29

**Authors:** Eurosurveillance editorial team

**Affiliations:** 1 European Centre for Disease Prevention and Control (ECDC), Stockholm, Sweden

**Keywords:** infectious diseases, public health, Europe

**Healthcare-associated infections and antibiotic resistance**

Meticillin-resistant Staphylococcus aureus 2016

EPI-NEWS 23, 2017

7 June 2017, Denmark

http://www.ssi.dk/Aktuelt/Nyhedsbreve/EPI-NYT/2017/Uge%2023%20-%202017.aspx

Laboratory surveillance of *Klebsiella* spp. bacteraemia in England, Wales and Northern Ireland: 2016

Health Protection Report; 11(18)

22 May 2017, United Kingdom

https://www.gov.uk/government/uploads/system/uploads/attachment_data/file/615375/hpr1817_klbsll.pdf

Laboratory surveillance of *Escherichia coli* bacteraemia in England, Wales and Northern Ireland: 2016 Health Protection Report; 11(18)

22 May 2017, United Kingdom

https://www.gov.uk/government/uploads/system/uploads/attachment_data/file/615340/hpr1817_ecoli.pdf

Laboratory surveillance of *Enterococcus* spp. bacteraemia in England, Wales and Northern Ireland: 2016 Health Protection Report; 11(15)

21 April 2017, United Kingdom

https://www.gov.uk/government/uploads/system/uploads/attachment_data/file/610226/hpr1517_ntrcccs.pdf

Laboratory surveillance of *Acinetobacter* spp. bacteraemia in England, Wales and Northern Ireland: 2016 Health Protection Report; 11(15)

21 April 2017, United Kingdom

https://www.gov.uk/government/uploads/system/uploads/attachment_data/file/610232/hpr1517_cntbctr.pdf

*Mycobacterium chimaera* in patients with a history of open heart surgery

Epi-Insight 2017;18(4)

April 2017, Ireland

http://ndsc.newsweaver.ie/epiinsight/15w224cjc8b10gkzp9yxn5?a=1&p=51673459&t=17517774

**Food- and waterborne diseases**

Outbreak of salmonellosis in North Dublin

Epi-Insight 2017;18(6)

June 2017, Ireland

http://ndsc.newsweaver.ie/epiinsight/1bcwq2qyang1qsl0rhv973?a=2&p=51918507&t=17517804

Gastro-intestinal and foodborne infections: viral pathogens, *Listeria*, *Shigella* and *Yersinia*

HPS Weekly Report 2017; 51(20)

23 May 2017, Scotland

http://www.hps.scot.nhs.uk/documents/ewr/pdf2017/1720.pdf

Reporting trends of shigellosis in the Netherlands, 1988-2015

Infectieziekten Bulletin 2017; 28(4)

24 April 2017, the Netherlands

http://www.rivm.nl/dsresource?objectid=a2fa844f-ade0-4e00-8314-91d54dbfd79d&type=pdf&disposition=inline

**Hepatitides**

Laboratory reports of hepatitis A and C (England and Wales): October to December 2016 Health Protection Report; 11(16)

28 April 2017, United Kingdom

https://www.gov.uk/government/uploads/system/uploads/attachment_data/file/611729/hpr1617_hep_AC.pdf

Hepatitis A vaccination gaps in men who have sex with men

Epidemiologisches Bulletin 13, 2017

30 March 2017, Germany

https://www.rki.de/DE/Content/Infekt/EpidBull/Archiv/2017/Ausgaben/13_17.pdf?__blob=publicationFile

**Vaccine-preventable diseases**

Laboratory reports of *Haemophilus influenzae* by age group and serotype, England and Wales: January to March 2017 (2016)

Health Protection Report; 11(19)

26 May 2017, United Kingdom

https://www.gov.uk/government/uploads/system/uploads/attachment_data/file/616220/hpr1917_hib.pdf

Laboratory confirmed cases of measles, mumps and rubella, England: January to March 2017

Health Protection Report; 11(19)

26 May 2017, United Kingdom

https://www.gov.uk/government/uploads/system/uploads/attachment_data/file/616215/hpr1917_mmr.pdf

Pertussis vaccination programme for pregnant women update: vaccine coverage in England, January to March 2017

Health Protection Report; 11(19)

26 May 2017, United Kingdom

https://www.gov.uk/government/uploads/system/uploads/attachment_data/file/616198/hpr1917_prntl-prtsssVC.pdf

Measles outbreaks in Ireland and other countries - both children and adults at risk

Epi-Insight 2017;18(5)

May 2017, Ireland

http://ndsc.newsweaver.ie/epiinsight/1s68n404xe91qsl0rhv973?a=1&p=51784315&t=17517774

Vaccine coverage estimate for the GP based catch-up meningococcal ACWY (MenACWY) immunisation programme for school leavers (becoming 18 before 31 August 2016) in England, cumulative data to the end of March 2017

Health Protection Report; 11(16)

28 April 2017, United Kingdom

https://www.gov.uk/government/uploads/system/uploads/attachment_data/file/612738/hpr1617_menACWY-vc2.pdf

Epidemiology of measles in Germany 2017

Epidemiologisches Bulletin 16, 2017

20 April 2017, Germany

https://www.rki.de/DE/Content/Infekt/EpidBull/Archiv/2017/Ausgaben/16_17.pdf?__blob=publicationFile

Pertussis 2016

EPI-NEWS 14, 2017

5 April 2017, Denmark

http://www.ssi.dk/Aktuelt/Nyhedsbreve/EPI-NYT/2017/Uge%2014%20-%202017.aspx

Measles

Epi-Ice 10(2), 2017

April 2017, Iceland

http://www.landlaeknir.is/servlet/file/store93/item32291/EPI-ICE_April_2017.pdf

Vaccines and vaccination: truth and fallacies

Not Ist Super Sanità 2017;30(3)

March 2017, Italy

http://www.iss.it/binary/publ/cont/ONLINE_MARZO_2017.pdf

**Respiratory diseases**

Influenza season 2016/2017

EPI-NEWS 24, 2017

14 June 2017, Denmark

http://www.ssi.dk/Aktuelt/Nyhedsbreve/EPI-NYT/2017/Uge%2024%20-%202017.aspx

Group A streptococcal infections: Third update on seasonal activity, 2016/17

Health Protection Report; 11(18)

12 May 2017, United Kingdom

https://www.gov.uk/government/uploads/system/uploads/attachment_data/file/613906/hpr1717_SF-GAS.pdf

Unraveling a tuberculosis cluster

Vlaams Infectieziektebulletin; 1/2017

April 2017, Belgium

https://www.zorg-en-gezondheid.be/sites/default/files/atoms/files/tb-ontrafeling-C.dehollogne.pdf

Contact tracing after the death of a patient with XDR-tuberculosis on an airplane, Germany 2013

Epidemiologisches Bulletin 13, 2017

30 March 2017, Germany

https://www.rki.de/DE/Content/Infekt/EpidBull/Archiv/2017/Ausgaben/13_17.pdf?__blob=publicationFile

**Sexually transmitted diseases**

Syphilis 2016

EPI-NEWS 21/22, 2017

31 May 2017, Denmark

http://www.ssi.dk/Aktuelt/Nyhedsbreve/EPI-NYT/2017/Uge%2021-22%20-%202017.aspx

Gonorrhoea 2016

EPI-NEWS 18, 2017

3 May 2017, Denmark

http://www.ssi.dk/Aktuelt/Nyhedsbreve/EPI-NYT/2017/Uge%2018%20-%202017.aspx

Sexually transmitted diseases

Epi-Ice 2017;10(2)

April 2017, Iceland

http://www.landlaeknir.is/servlet/file/store93/item32291/EPI-ICE_April_2017.pdf

Sexually transmitted infections and HIV in Ireland, 2016: provisional data

Epi-Insight 2017;18(4)

April 2017, Ireland

http://ndsc.newsweaver.ie/epiinsight/s9b8h9tdfmi10gkzp9yxn5?a=2&p=51673455&t=17517804

**Zoonoses and vector-borne diseases**

Surveillance of chikungunya, dengue and Zika virus infection in mainland France, 2016

Bulletin épidémiologique hebdomadaire, 12

30 May 2017, France

http://invs.santepubliquefrance.fr/beh/2017/12/pdf/2017_12.pdf

Zoonotic infections with Mycobacterium tuberculosis in German livestock

Epidemiologisches Bulletin 20, 2017

18 May 2017, Germany

https://www.rki.de/DE/Content/Infekt/EpidBull/Archiv/2017/Ausgaben/20_17.pdf?__blob=publicationFile

Common animal associated infections quarterly report (England and Wales) – first quarter 2017 Health Protection Report; 11(17)

12 May 2017, United Kingdom

https://www.gov.uk/government/uploads/system/uploads/attachment_data/file/614121/hpr1717_zoos2.pdf

Psittacosis 2016

EPI-NEWS 19, 2017

10 May 2017, Denmark

http://www.ssi.dk/Aktuelt/Nyhedsbreve/EPI-NYT/2017/Uge%2019%20-%202017.aspx

Tick-borne encephalitis, a new disease in the Netherlands?

Infectieziekten Bulletin 2017; 28(4)

24 April 2017, the Netherlands

http://www.rivm.nl/dsresource?objectid=de36ea65-7b14-45ac-9e91-3629d14fc0f4&type=pdf&disposition=inline

Special edition: Leptospirosis in the French overseas regions and departments

Bulletin épidémiologique hebdomadaire, 8-9

4 April 2017, France

http://invs.santepubliquefrance.fr/beh/2017/8-9/pdf/2017_8-9.pdf

Bovine tuberculosis in Limburg

Vlaams Infectieziektebulletin; 1/2017

April 2017, Belgium

https://www.zorg-en-gezondheid.be/sites/default/files/atoms/files/VIB%202017-1%20-%20Rundertuberculose%20in%20Limburg.pdf

**Other**

HPS report on laboratory-confirmed travel-related infections in Scotland during 2016

HPS Weekly Report 2017; 51(18)

9 May 2017, Scotland

http://www.hps.scot.nhs.uk/documents/ewr/pdf2017/1718.pdf

TRAVAX Outbreak Index 2016: Diseases of Public and Travel Health Risk

HPS Weekly Report 2017; 51(17)

2 May 2017, Scotland

http://www.hps.scot.nhs.uk/documents/ewr/pdf2017/1717.pdf

